# Supplementary material for: Translational pharmacology of an inhaled small molecule αvβ6 integrin inhibitor for idiopathic pulmonary fibrosis
Source: Nat Commun. 2020 Sep 16;11:4659. doi: 10.1038/s41467-020-18397-6 (PMC7494911; doi:10.1038/s41467-020-18397-6)
Supplement: Supplementary file 1 — Supplementary Information [file 41467_2020_18397_MOESM1_ESM.pdf]

Title: Translational pharmacology of an inhaled small molecule αvβ6 integrin inhibitor for idiopathic pulmonary fibrosis

Authors: Alison E. John[1], Rebecca H. Graves[2], K. Tao Pun[2], Giovanni Vitulli[2], Ellen J. Forty[3], Paul F. Mercer[3], Josie L. Morrell[2], John W. Barrett[2], Rebecca F. Rogers[2], Maryam Hafeji[2], Lloyd I. Bibby[2], E. Gower[2], Valerie S. Morrison[2], Yim Man[2], James A. Roper[2], Jeni C. Luckett[4], Lee A. Borthwick[5], Ben S. Barksby[5], Rachel A. Burgoyne[5], Rory Barnes[5], Joelle Le[6], David J. Flint[7], Susan Pyne[7], Anthony Habgood[1], Louise A. Organ[1], Chitra Joseph[1], Rochelle C. Edwards-Pritchard[1], Toby M. Maher[8], Andrew J. Fisher[5,9], Natasja Stæhr Gudmann[10], Diana J. Leeming[10], Rachel C. Chambers[3], Pauline T. Lukey[2], Richard P. Marshall[2], Simon J.F. Macdonald[2], R. Gisli Jenkins[1*], Robert J. Slack[2].

Affiliations:

[1]Respiratory Medicine NIHR Biomedical Research Centre, University of Nottingham, Nottingham, UK.

[2]Fibrosis DPU, Respiratory TAU, GlaxoSmithKline, Stevenage, Hertfordshire, UK.

[3]Centre for Inflammation and Tissue Repair, University College London, London, UK.

[4]Radiological Sciences, University of Nottingham, Nottingham, UK

[5]Fibrosis Research Group, Newcastle University Biosciences Institute and Newcastle University Translational and Clinical Research Institute, Newcastle upon Tyne, UK.

[6]Drug Design and Selection - Molecular Design, Respiratory TAU, GlaxoSmithKline, Stevenage, Hertfordshire, UK.

[7]Strathclyde Institute of Pharmacy and Biomedical Sciences, University of Strathclyde, Glasgow, UK.

[8]NIHR Respiratory Clinical Research Facility, Royal Brompton Hospital, London, UK and Fibrosis Research Group, National Heart and Lung Institute, Imperial College, London, UK.

[9]Institute of Transplantation, Freeman Hospital, Newcastle Upon Tyne Hospitals NHS, Foundation Trust, Newcastle upon Tyne, UK.

[10]Nordic Bioscience A/S, Biomarkers and Research, Herlev Hovedgade 205-207, DK-2730 Herlev, Denmark

*Corresponding author. E-mail: gisli.jenkins@nottingham.ac.uk

Supplementary Methods

Computational Modelling

The αvβ6 homology model was developed using Molecular Operating Environment (Chemical Computing Group, QC, Canada) and the αvβ3 X-ray structure 1L5G *(26)* (closed form of integrin) as template. During lead optimization, compounds were modelled into the binding site using Maestro (Schrödinger). The binding site for docking was defined using the centroid of the co-crystallized cyclic peptide coordinates (from 1L5G) as input for Glide's Grid receptor generation function and Glide with Standard Precision docking was used for binding mode prediction (1-3). The preferred poses of docked compounds were selected using criteria of favorable protein-ligand interactions, acceptable ligand geometry, good shape and surface complementarity with the αvβ6 receptor.

Pharmacokinetic Analysis

Sample analysis

Blood samples from all in vivo studies were diluted 1:1 with water or 0.02% phosphoric acid and stored at -20°C prior to analysis. Samples were thawed and mixed with acetonitrile containing internal standard and centrifuged at 1500 *g* for 10 min. Supernatants were evaporated to dryness under nitrogen and reconstituted in acetonitrile:water 10:90 (v/v). The concentration of GSK3008348 in blood was determined by liquid chromatography-tandem mass spectrometry (LC-MS/MS). Lung tissue samples were weighed and homogenized in water, using a Precellys (Stretton Scientific Ltd, Derbyshire, UK) 7 ml tube with ceramic beads. Aliquots of the homogenates were mixed with acetonitrile containing internal standard and centrifuged. Supernatants were evaporated to dryness under nitrogen and reconstituted in acetonitrile:water 10:90 (v/v). The

concentration of GSK3008348 in tissue homogenate was determined by LC-MS/MS. All samples from in vitro/ex vivo studies were extracted by protein precipitation followed by centrifugation, unless stated otherwise. In vitro permeability samples were quantified by LC-MS/MS cassette analysis using a 5-point calibration with appropriate dilution of the samples.

Nebulized pharmacokinetics in the rat

Pharmacokinetics by the nebulized route was investigated in the female Wistar Han rat (n=3, body weight 270-295 g) housed in standard holding cages and maintained in a controlled environment with free access to food and water. The study was conducted to generate systemic pharmacokinetics and a lung retention time course for GSK3008348 after nebulization in an ADG inhalation tower (ADG Developments Ltd., Essex, UK). On the day of the nebulized PK study, fourteen rats (280-330 g) were placed in a restrainer and randomly attached to ports on an inhalation tower prior to the start of the nebulization (using an LC® Sprint jet nebulizer (PARI Respiratory Equipment, Inc., VA, Canada)). All rats were exposed for 10 min to an aerosol generated from a solution containing GSK3008348 at 10 mg/ml, formulated in 100% saline adjusted to pH 7.0. The target dose level for the study was 1 mg/kg, but subsequent filter analysis allowed the estimation of the actual dose level as 0.227 mg/kg. Serial blood samples from 4 rats were collected up to 7 h from the beginning of the nebulization from a temporary cannula in the tail vein into a heparinized microtainer. All rats in the study were culled (n=2 rats/timepoint, up to 12 h) with an overdose of pentobarbitone. A terminal blood sample was collected for all rats from a severed jugular vein into a heparinized microtainer and the lungs were removed and kept at 4ºC. Individual aliquots of blood were transferred directly into micronics tubes containing an equal volume of sterile water and stored frozen, together with the lung tissue, and stored at -20ºC prior to analysis.

Intranasal pharmacokinetics in the mouse

Pharmacokinetics by the i.n. route was investigated in female C57Bl/6J mice (n=12, body weight 19-22 g) housed in standard holding cages and maintained in a controlled environment with free access to food and water. GSK3008348 was dosed by the i.n. route, together ('cassette' study design) with another discovery compound and a reference compound for cross-studies normalization and to confirm dosing success, with each compound dosed at 1 mg/kg. GSK3008348 was administered as a clear solution obtained by pooling equal volumes of individual solutions for each of the three compounds, dissolved in 100 % saline at 1.5 mg/ml. After applying a light isoflurane anaesthesia (3-4%), the mice were lightly scuffed to create a vertical line from the nose to the lung. A fixed volume of 50 µl was dripped between the nostrils and allowed to be inhaled. Animals were returned to the home cage and propped up on their back to recover. Lungs were harvested (n=2 per timepoint) on a time course 5 min to 12 h after the compound administration and stored at -20°C pending analysis. Blood samples were also obtained over the same time course using a composite design, sampling from the tail vein or by terminal cardiac puncture with all samples taken into heparinized containers. Individual aliquots of blood were transferred directly into micronics tubes containing an equal volume of sterile water and stored frozen at -20°C prior to analysis.

In vitro and ex vivo tissue binding

The in vitro binding of GSK3008348 to lung homogenate was measured in mouse and human lung tissue by rapid equilibrium dialysis at the nominal concentration of 1 µg/ml (in diluted homogenate). For each in vitro binding study approximately 1 g (accurate weight recorded used for subsequent homogenate dilution) of mouse or human lung tissue was weighed out and placed into a 7 ml Precellys homogenizing tube prefilled with CK28 ceramic beads. 4 ml of buffer (PBS

with 100 mM sodium phosphate and 150 mM sodium chloride, pH 6.9 -7.2) were added before being homogenized. The resulting homogenate was further diluted with buffer to create a 1:10 diluted homogenate, spiked at 1 μg/ml GSK3008348 and incubated for 4 hours at 37°C, with aliquots removed at the beginning and end of the incubation period to assess the stability of the compound in tissue along with the binding. All buffer and lung homogenate samples (50μl) were matrix matched by diluting 2-fold with either lung homogenate or buffer before being taken for analysis. The resultant samples were extracted by protein precipitation and the lung tissue binding of GSK3008348 determined by peak area ratio from the LC-MS/MS analysis.

The binding of GSK3008348 to mouse lung homogenate was also measured ex vivo, using the lungs from an in vivo study (sub-cutaneous administration to the male C57Bl/6J mouse at 15 mg/kg/day by osmotic mini-pumps). Experimental conditions for the preparation of the homogenate, the rapid equilibrium dialysis, the LC-MS/MS analysis and the processing of the results were as described above.

In vitro passive permeability

The permeability of GSK3008348 across an MDCK-MDR1 cell monolayer was measured in the presence of an efflux inhibitor (GF120918 (4) in Hanks' Balanced Salt Solution (pH 7.4). Incubations were carried out in an atmosphere of 5% CO_2 with a relative humidity of 95% at 37°C for 60 min. Apical and basolateral samples were diluted for analysis by LC-MS/MS.

Data analysis

Pharmacokinetic parameters for all in vivo studies were derived from concentration *versus* time profiles using WinNonlin v6.3 (Certara, Princeton, NJ, USA). Non-compartmental methods were used to obtain estimates of pharmacokinetic parameters for each animal and mean values obtained

by averaging the individual parameters. The data were fitted with two-compartment models with first-order elimination rates.

Supplementary References

1. R.A. Friesner, R.B. Murphy, M.P. Repasky, L.L. Frye, J.R. Greenwood, T.A. Halgren, P.C. Sanschagrin, D.T. Mainz, Extra precision Glide: docking and scoring incorporating a model of hydrophobic enclosure for protein-ligand complexes. *J. Med. Chem.* **49**, 6177–6196 (2006).

2. R.A. Friesner, J.L. Banks, R.B. Murphy, T.A. Halgren, J.J. Klicic, D.T.Mainz, M.P. Repasky, E.H. Knoll, D.E. Shaw, M. Shelley, J.K. Perry, P. Francis, P.S. Shenkin, Glide: a new approach for rapid, accurate docking and scoring. 1. method and assessment of docking accuracy. *J. Med. Chem.* **47**, 1739–1749 (2004).

3. T.A. Halgren, R.B. Murphy, R.A. Friesner, H.S. Beard, L.L. Frye, W.T. Pollard, J.L. Banks, Glide: a new approach for rapid, accurate docking and scoring. 2. enrichment factors in database screening. *J. Med. Chem.* **47**, 1750–1759 (2004).

4. S.P. Letrent, G.M. Pollack, K.R. Brouwer, K.L. Brouwer, Effect of GF120918, a potent P-glycoprotein inhibitor, on morphine pharmacokinetics and pharmacodynamics in the rat. *Pharm. Res.* **15**, 599-605 (1998).

Supplementary Figures

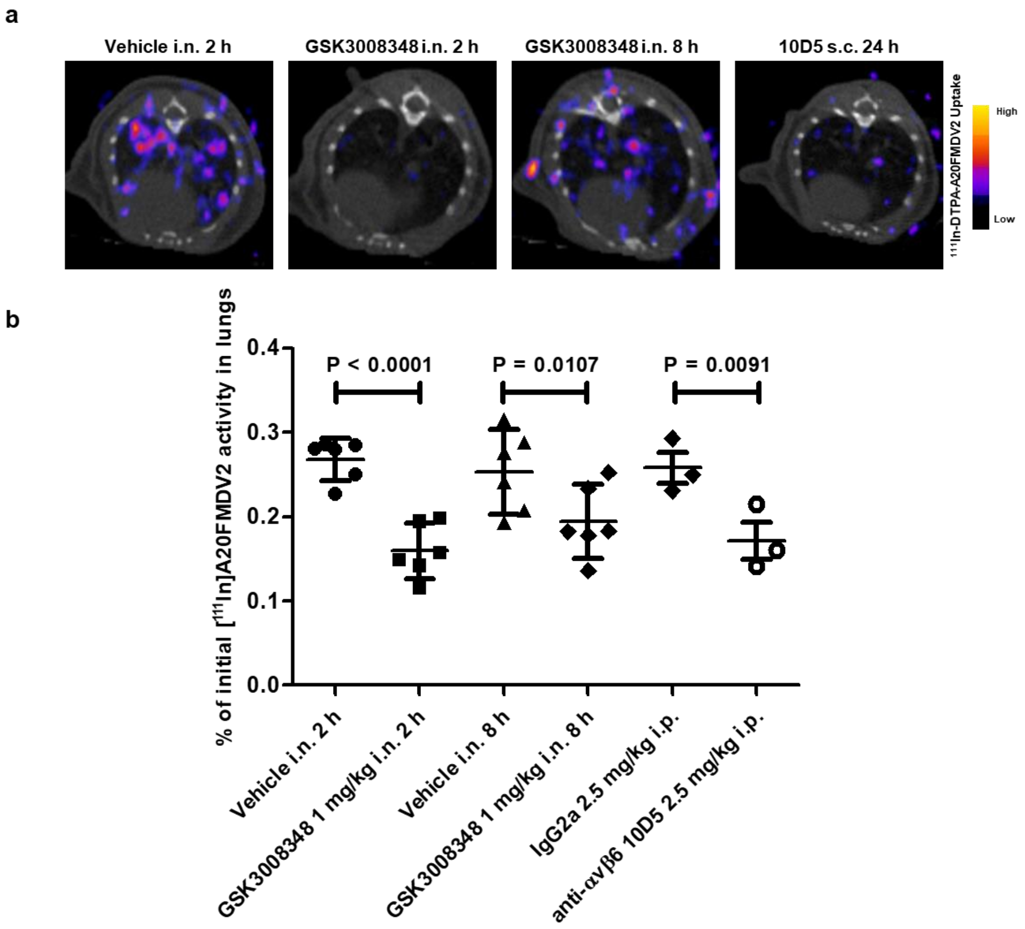

Supplementary Figure 1. Time-dependent in vivo engagement of αvβ6 by GSK3008348.

a Representative axial SPECT/CT scans of thorax showing binding of the selective αvβ6 integrin nanoSPECT-CT ligand [^{111}In]A20FMDV2 in naïve mice 2 h and 8 h post-i.n. dosing with GSK3008348 or 24 h after the administration of 10D5 antibody. **b** Quantification of [^{111}In]A20FMDV2 binding within the lungs in response to treatment (mean ± SEM; n=3-6 animals per group; statistical analysis ANOVA with Fisher's LSD post-test). Source data are provided as a Source Data file.

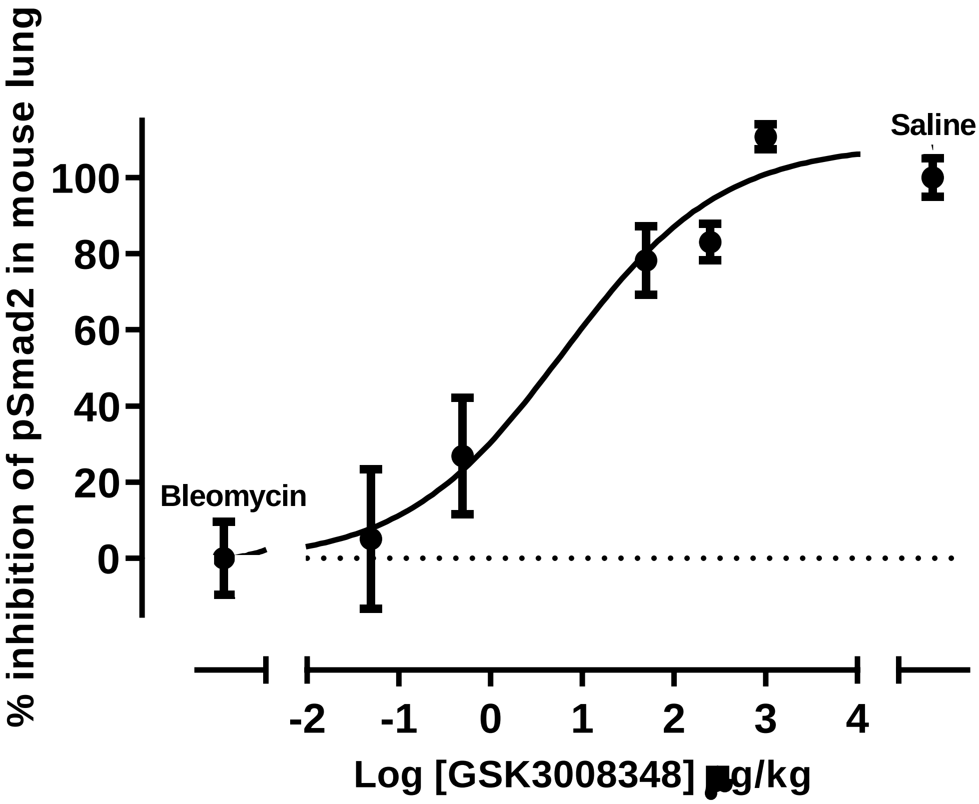

Supplementary Figure 2. Dose-dependent effect of GSK3008348 on pSmad2 in vivo

Dose-dependent inhibition of lung tissue pSmad2 levels in bleomycin-treated mice 2 h post-i.n. dosing of GSK3008348 (mean ± SEM; n=10-20 animals per group). Source data are provided as a Source Data file

Supplementary Table 1. Total and predicted free lung concentrations of GSK3008348A

Time point (h)	Mouse Number	Total Lung [GSK3008348] ng/lung	Predicted Free Lung [GSK3008348] ng/lung
0.083	1	<2*	<0.06
	2	290	9.3
0.5	3	87.5	2.8
	4	72.1	2.3
2	5	<2	<0.06
	6	6.94	0.2
4	7	<2	<0.06
	8	<2	<0.06
7	9	<2	<0.06
	10	<2	<0.06
12	11	<2	<0.06
	12	<2	<0.06

Male C57BL/6 mouse were dosed intransally at 1 mg/kg and lung samples collected at timepoints up to 12 h post dosing. The lower limit of quantitation *via* reverse phase HPLC/MS/MS was 2 ng/lung with predicted free levels of GSK3008348 calculated using a fraction unbound in mouse lung tissue of 3.2% (see supplementary methods section above). *Mis-dosed animal.

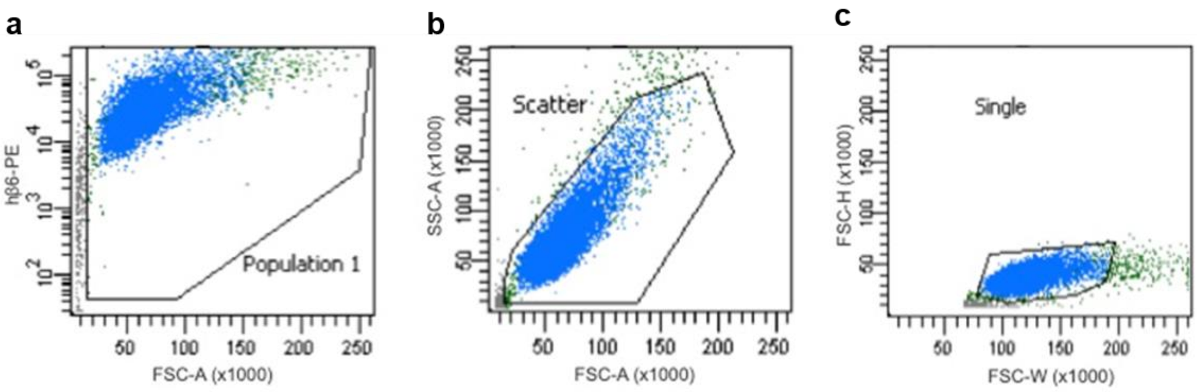

Supplementary Figure 3. NHBE and SAE cells flow cytometry gating strategy.

a Whole cells were distinguished from debris by their greater forward scatter characteristics ("Population 1" within gate). **b** Cells were verified as a single population by forward- and side-scatter comparison ("Scatter" within gate) with further removal of debris. **c** A single cell population was selected by width and height comparison of the forward-scatter ("Single" within gate). Events that satisfy all three gated populations (coloured blue in each scatter plot) were designated as whole single cells and the mean fluorescence intensity (MFI) of antibody signal for these cells measured with the fluorescence quantified on at least 5,000 cells. FSC-A, forward scatter area; FSC-H, forward scatter height; FSC-W, forward scatter width; SSC-A, side scatter area.
