# Supplementary material for: Translational pharmacology of an inhaled small molecule αvβ6 integrin inhibitor for idiopathic pulmonary fibrosis
Source: Nat Commun. 2020 Sep 16;11:4659. doi: 10.1038/s41467-020-18397-6 (PMC7494911; doi:10.1038/s41467-020-18397-6)
Supplement: Supplementary file 3 — Reporting Summary [file 41467_2020_18397_MOESM3_ESM.pdf]

nature research

Corresponding author(s): Gisli Jenkins, NCOMMS-18-34532A-Z

Reporting Summary

Nature Research wishes to improve the reproducibility of the work that we publish. This form provides structure for consistency and transparency in reporting. For further information on Nature Research policies, see Authors & Referees and the Editorial Policy Checklist.

Statistical parameters

When statistical analyses are reported, confirm that the following items are present in the relevant location (e.g. figure legend, table legend, main text, or Methods section).

n/a	Confirmed	
[ ]	[x]	The exact sample size (*n*) for each experimental group/condition, given as a discrete number and unit of measurement
[x]	[ ]	An indication of whether measurements were taken from distinct samples or whether the same sample was measured repeatedly
[ ]	[x]	The statistical test(s) used AND whether they are one- or two-sided<br>*Only common tests should be described solely by name; describe more complex techniques in the Methods section.*
[x]	[ ]	A description of all covariates tested
[x]	[ ]	A description of any assumptions or corrections, such as tests of normality and adjustment for multiple comparisons
[ ]	[x]	A full description of the statistics including central tendency (e.g. means) or other basic estimates (e.g. regression coefficient) AND variation (e.g. standard deviation) or associated estimates of uncertainty (e.g. confidence intervals)
[ ]	[x]	For null hypothesis testing, the test statistic (e.g. *F*, *t*, *r*) with confidence intervals, effect sizes, degrees of freedom and *P* value noted<br>*Give P values as exact values whenever suitable.*
[x]	[ ]	For Bayesian analysis, information on the choice of priors and Markov chain Monte Carlo settings
[x]	[ ]	For hierarchical and complex designs, identification of the appropriate level for tests and full reporting of outcomes
[x]	[ ]	Estimates of effect sizes (e.g. Cohen's *d*, Pearson's *r*), indicating how they were calculated
[ ]	[x]	Clearly defined error bars<br>*State explicitly what error bars represent (e.g. SD, SE, CI)*

Our web collection on statistics for biologists may be useful.

Software and code

Policy information about availability of computer code

Data collection	No software was used.
Data analysis	All figures were generated in Prism 7.0 (GraphPad Software, San Diego, CA, USA) with concentration response curves fitted using the standard Prism 7.0 equation 'log(inhibitor) vs. response -- Variable slope (four parameters)') and saturation binding fitted using the standard Prism 7.0 equation 'One site -- Specific binding with Hill slope'. Statistical analyses were also completed using Prism 7.0 for in vitro studies and R version 3.4 for in vivo/ex vivo studies.

For manuscripts utilizing custom algorithms or software that are central to the research but not yet described in published literature, software must be made available to editors/reviewers upon request. We strongly encourage code deposition in a community repository (e.g. GitHub). See the Nature Research guidelines for submitting code & software for further information.

Data

Policy information about availability of data

All manuscripts must include a data availability statement. This statement should provide the following information, where applicable:

- Accession codes, unique identifiers, or web links for publicly available datasets
- A list of figures that have associated raw data
- A description of any restrictions on data availability

The data that support the findings of this study are available from the corresponding author upon reasonable request.

Field-specific reporting

Please select the best fit for your research. If you are not sure, read the appropriate sections before making your selection.

- [x] Life sciences
- [ ] Behavioural & social sciences
- [ ] Ecological, evolutionary & environmental sciences

For a reference copy of the document with all sections, see nature.com/authors/policies/ReportingSummary-flat.pdf

Life sciences study design

All studies must disclose on these points even when the disclosure is negative.

Sample size

Mice were randomized into treatment groups and for initial studies sample size was selected based on experience of the model. In subsequent studies power analysis was completed on the previous studies data to determine sample size.

Data exclusions

No data were excluded from the analyses.

Replication

All in vitro experiments were replicated successfully at least 4 times on separate test occasions with no failed studies reported. For in vivo experiments the effect of GSK3008348 on SPECT imaging and Smad2 levels were successfully reproduced at least twice with no failed studies reported (see supplementary data).

Randomization

Animals were randomly assigned to cages by an animal technician and each cage was designated at random to receive bleomycin or saline by the primary researcher. Mice were treated with saline/bleomycin as designated, ear notched for identification and assigned a mouse ID number. For drug dosing studies, on the day of dosing all animals remaining in the study were randomised to treatment groups using computer generated randomisation (Excel).

Blinding

All samples were identified only by a mouse ID number and researchers performing sample processing and analysis were blinded to the treatment groups. Samples were unblinded only after sample analysis was completed.

Reporting for specific materials, systems and methods

Materials & experimental systems

n/a	Involved in the study	
x		Unique biological materials
	x	Antibodies
	x	Eukaryotic cell lines
x		Palaeontology
	x	Animals and other organisms
x		Human research participants

Methods

n/a	Involved in the study	
x		ChIP-seq
	x	Flow cytometry
x		MRI-based neuroimaging

Antibodies

Antibodies used	For flow cytometry PE-conjugated mouse monoclonal anti-human integrin beta-6 (clone #437211, catalogue #FAB4155P, lot #AAPP01 from R&D Systems, Minneapolis, MN, USA). For immunocytochemistry sheep polyclonal anti-human integrin β6 (clone accession # P18564, catalogue #AF4155 from R&D Systems, Minneapolis, MN, USA). For functional integrin blocking studies monoclonal anti-human integrin αvβ6 (clone 10D5, catalogue #MAB2077Z from Merck Millipore, Billerica, MA, USA).
Validation	PE-conjugated mouse monoclonal anti-human integrin beta-6 (clone #437211, catalogue #FAB4155P, lot #AAPP01 from R&D Systems, Minneapolis, MN, USA) - Pub Med ID for antibody validation: 22659470, 26734728.<br>Sheep polyclonal anti-human integrin β6 (catalogue #AF4155 from R&D Systems, Minneapolis, MN, USA). Manufacturers information: Detects human integrin beta-6 in the direct ELISAs and Western blots. In direct ELISAs, approximately 35% cross-reactivity with recombinant mouse integrin beta-6 is observed and less than 5% cross-reactivity with recombinant human integrin beta-3 and integrin beta-5 is observed. Immunogen: mouse myeloma cell line NS0-derived recombinant human integrin beta-6 (Gly22-Asn707, accession #P18564).<br>Monoclonal anti-human integrin αvβ6 (clone 10D5, catalogue #MAB2077Z from Merck Millipore, Billerica, MA, USA) - Pub Med ID for antibody validation 24478423, 24150233, 23275294, 22455378, 21774545, 21079788, 19088289, 18459483, 18566227, 17575158.

Eukaryotic cell lines

Policy information about cell lines

Cell line source(s)	Normal human bronchial epithelial (NHBE) cells obtained from Lonza (Lonza Group Ltd, Basel, Switzerland).<br>Small Airway Epithelial (SAEC) cells obtained from Lonza (Lonza Group Ltd, Basel, Switzerland). IPF SAECs were obtained from Collaborators at University of
Authentication	Routine characterization of NHBE cells and SAECs by morphological observation throughout serial passages.
Mycoplasma contamination	Cell lines tested negative for mycoplasma contamination.
Commonly misidentified lines (See ICLAC register)	Study did not use commonly misidentified lines.

Animals and other organisms

Policy information about studies involving animals; ARRIVE guidelines recommended for reporting animal research

Laboratory animals	Male C57BL/6 mice (6-12 weeks old from Charles River, Kent, UK) were acclimatized for 5-7 days before undergoing procedures and ranged from 16 to 28g at the time of study.
Wild animals	Study did not involve wild animals.
Field-collected samples	Study did not involve samples collected from the field.

Flow Cytometry

Plots

Confirm that:

- ☐ The axis labels state the marker and fluorochrome used (e.g. CD4-FITC).
- ☐ The axis scales are clearly visible. Include numbers along axes only for bottom left plot of group (a 'group' is an analysis of identical markers).
- ☐ All plots are contour plots with outliers or pseudocolor plots.
- ☐ A numerical value for number of cells or percentage (with statistics) is provided.

Methodology

Sample preparation	Normal human bronchial epithelial (NHBE) cells obtained from Lonza (Lonza Group Ltd, Basel, Switzerland). NHBE cells were maintained in culture following supplier guidelines in NHBE cell medium (bronchial epithelial growth medium (BEGM) containing 0.6 mmol/l MgCl2 supplemented with BEGM Clonetics SingleQuots (containing bovine pituitary extract, insulin, hydrocortisone, GA-1000 (consisting of 30 mg/ml gentamicin and 15 µg/ml amphotericin), retinoic acid, transferrin, triiodothyronine, epinephrine and human epidermal growth factor)) in 95:5% air:CO2 at 37°C). Cells were harvested when ~80% confluent using Accutase® then re-suspended in PBS, centrifuged at 300 g for 5 min prior to re-suspension in flow cytometry buffer (RPMI 1640 (without L-glutamine and phenol red) containing 10 mmol/l HEPES, 1% w/v bovine serum albumin and 2 mmol/l MgCl2). Cells were then counted on a NucleoCounter NC-3000 (ChemoMetec, Allerød, Denmark) and re-suspended to the required cell density.
Instrument	FACS Canto II (BD Biosciences, San Jose, CA, USA)

Software	BD FACS DivaTM version 6.1.3 software. Fluorescence-activated cell analyses histograms were plotted using FlowJo software (Tree Star Inc. Ashland, OR, USA).
Cell population abundance	Only cells lines ie single cell populations were analysed by flow cytometry
Gating strategy	Whole cells were distinguished from debris by their greater forward scatter characteristics (“Population 1” within gate). Cells were verified as a single population by forward- and side-scatter comparison (“Scatter” within gate) with further removal of debris. A single cell population was selected by width and height comparison of the forward-scatter (“Single” within gate). Events that satisfy all three gated populations were designated as whole single cells and the mean fluorescence intensity (MFI) of antibody signal for these cells measured with the fluorescence quantified on at least 5,000 cells.

☒ Tick this box to confirm that a figure exemplifying the gating strategy is provided in the Supplementary Information.
